# The complete digital workflow in fixed prosthodontics: a systematic review

**DOI:** 10.1186/s12903-017-0415-0

**Published:** 2017-09-19

**Authors:** Tim Joda, Fernando Zarone, Marco Ferrari

**Affiliations:** 1 Section for Digital Reconstructive Technology + Implant Dentistry [DiRecT + ID], Department of Reconstructive Dentistry, School of Dental Medicine, University of Bern, Freiburgstr. 7, 3010 Bern, Switzerland; 2 Department of Prosthodontics, School of Dental Medicine, University of Napoli, Naples, Italy; 3 Department of Prosthodontics & Dental Materials, School of Dental Medicine, University of Siena, Siena, Italy; 4 Department of Restorative Dentistry, School of Dental Medicine, University of Leeds, Leeds, UK; 5 Department of Reconstructive Dentistry, University Center for Dental Medicine, Basel, Switzerland

**Keywords:** Systematic review, Fixed prosthodontics, Tooth-bourne, Implant-supported, Complete digital workflow

## Abstract

**Background:**

The continuous development in dental processing ensures new opportunities in the field of fixed prosthodontics in a complete virtual environment without any physical model situations. The aim was to compare fully digitalized workflows to conventional and/or mixed analog-digital workflows for the treatment with tooth-borne or implant-supported fixed reconstructions.

**Methods:**

A PICO strategy was executed using an electronic (MEDLINE, EMBASE, Google Scholar) plus manual search up to 2016–09-16 focusing on RCTs investigating complete digital workflows in fixed prosthodontics with regard to economics or esthetics or patient-centered outcomes with or without follow-up or survival/success rate analysis as well as complication assessment of at least 1 year under function. The search strategy was assembled from MeSH-Terms and unspecific free-text words: {((“Dental Prosthesis” [MeSH]) OR (“Crowns” [MeSH]) OR (“Dental Prosthesis, Implant-Supported” [MeSH])) OR ((crown) OR (fixed dental prosthesis) OR (fixed reconstruction) OR (dental bridge) OR (implant crown) OR (implant prosthesis) OR (implant restoration) OR (implant reconstruction))} AND {(“Computer-Aided Design” [MeSH]) OR ((digital workflow) OR (digital technology) OR (computerized dentistry) OR (intraoral scan) OR (digital impression) OR (scanbody) OR (virtual design) OR (digital design) OR (cad/cam) OR (rapid prototyping) OR (monolithic) OR (full-contour))} AND {(“Dental Technology” [MeSH) OR ((conventional workflow) OR (lost-wax-technique) OR (porcelain-fused-to-metal) OR (PFM) OR (implant impression) OR (hand-layering) OR (veneering) OR (framework))} AND {((“Study, Feasibility” [MeSH]) OR (“Survival” [MeSH]) OR (“Success” [MeSH]) OR (“Economics” [MeSH]) OR (“Costs, Cost Analysis” [MeSH]) OR (“Esthetics, Dental” [MeSH]) OR (“Patient Satisfaction” [MeSH])) OR ((feasibility) OR (efficiency) OR (patient-centered outcome))}.

Assessment of risk of bias in selected studies was done at a ‘trial level’ including random sequence generation, allocation concealment, blinding, completeness of outcome data, selective reporting, and other bias using the Cochrane Collaboration tool. A judgment of risk of bias was assigned if one or more key domains had a high or unclear risk of bias. An official registration of the systematic review was not performed.

**Results:**

The systematic search identified 67 titles, 32 abstracts thereof were screened, and subsequently, three full-texts included for data extraction. Analysed RCTs were heterogeneous without follow-up. One study demonstrated that fully digitally produced dental crowns revealed the feasibility of the process itself; however, the marginal precision was lower for lithium disilicate (LS2) restorations (113.8 μm) compared to conventional metal-ceramic (92.4 μm) and zirconium dioxide (ZrO2) crowns (68.5 μm) (*p* < 0.05). Another study showed that leucite-reinforced glass ceramic crowns were esthetically favoured by the patients (8/2 crowns) and clinicians (7/3 crowns) (*p* < 0.05). The third study investigated implant crowns. The complete digital workflow was more than twofold faster (75.3 min) in comparison to the mixed analog-digital workflow (156.6 min) (p < 0.05). No RCTs could be found investigating multi-unit fixed dental prostheses (FDP).

**Conclusions:**

The number of RCTs testing complete digital workflows in fixed prosthodontics is low. Scientifically proven recommendations for clinical routine cannot be given at this time. Research with high-quality trials seems to be slower than the industrial progress of available digital applications. Future research with well-designed RCTs including follow-up observation is compellingly necessary in the field of complete digital processing.

**Electronic supplementary material:**

The online version of this article (10.1186/s12903-017-0415-0) contains supplementary material, which is available to authorized users.

## Background

The continuous development in the computer technology and dental processing ensures new opportunities in the field of fixed prosthodontics [1]. Traditionally, the standard treatment approach consisted of conventional impression techniques and stone casts for the manufacturing of acrylic- and porcelain-fused-to-metal reconstructions using the lost-wax-technique. In contrast, computerized engineering technology is related with consistent precision and reproducible production results in a streamlined work process with reduced manpower [2, 3].

The establishment of CAD/CAM-technology has been the game changer for the production of tooth-borne and implant-supported monolithic fixed dental prostheses (FDP) by means of digitally on-screen designing with dental software applications, and secondary computer-assisted production with rapid prototyping procedures, such as milling or 3D–printing, in a virtual environment without any physical model situations [4].

Several companies offer various computerized software applications and technical devices, and the dental team of clinician and technician has to choose how and when to proceed digitally or stay conventionally [5]. The truth in dental business reveals: there is neither the pure classical pathway nor a fully digital workflow [6]. Single digital work steps infiltrate the proven goldstandard approach [7]. Changes are growing in the field of prosthodontic treatment effecting impressions-taking procedures, which are more and more replaced by intraoral scanning (IOS) as well as the CAD/CAM-production of anatomically full-contour restorations or frameworks combined with CAD-on veneering. The result of this evolution is a mixed analog-digital workflow – combining best of both techniques [8].

In general, only a few technical reports have analysed digital workflows in fixed prosthodontics. The focus was limited to in-vitro studies investigating laboratory precision or clinical case series concentrating on single treatment steps, such as IOS compared to conventional impression-taking procedures [9–11].

The scientific validation and evidence for the clinical and technical feasibility, the biological (long-term) outcomes, and economic analyses of complete digital workflows is crucial to understand the impact of the actual digitalization trend on modifying well-established conventional protocols in fixed prosthodontics [12]. Especially, the following questions arise:

- What benefits do complete digital treatment concepts offer in the production of FDPs; and at what quality level compared to the goldstandard approach in a conventional pathway?
- Moreover, what are the economic outcomes in a fully digitalized workflow?

Today, no systematic review is available, which investigated complete digital workflows in prosthodontics. Therefore, the aim of this literature is to compare fully digitalized workflows to conventional and/or mixed analog-digital workflows for the treatment with tooth-borne and implant-supported fixed reconstructions. This systematic review followed the PRISMA statement (http://prisma-statement.org/).

## Methods

### Search strategy and study selection

Based on the PICO criteria, a search strategy was developed and executed using an electronic search. The PICO question was formulated as follows: “Is a complete digital workflow with intraoral optical scanning (IOS) plus virtual design plus monolithic restoration for patients receiving prosthodontic treatments with (A) tooth-borne or (B) implant-supported fixed reconstructions comparable to conventional or mixed analog-digital workflows with conventional impression and/or lost-wax-technique and/or framework and veneering in terms of feasibility in general or survival/success-analysis including complication assessment with a minimum follow-up of one year or economics or esthetics or patient-centered factors?”

A MEDLINE (PubMed) and EMBASE search, including grey literature by means of Google Scholar, up to 2016–09-16 was then performed using the following search terms. Search terms were grouped into categories for “Problem” – “Intervention” – “Control” – “Outcome”. The search strategy was assembled from a combination of qualified Medical Subject Headings (MeSH-Terms) as well as unspecific free-text words in simple or multiple conjunctions as presented in Table 1:

**Table 1:** Overview of the electronic search strategy including timeline and P-I-C-O definition for study selection

Timeline	Up to 2016–09-16
P – I – C – O	Problem {(“Dental Prosthesis” [MeSH]) OR (“Crowns” [MeSH]) OR (“Dental Prosthesis, Implant-Supported” [MeSH] OR (“Crowns, Implant-Supported” [MeSH]) OR (crown) OR (fixed dental prosthesis) OR (fixed reconstruction) OR (dental bridge) OR (implant crown) OR (implant prosthesis) OR (implant restoration) OR (implant reconstruction)} AND
	Intervention {(“Computer-Aided Design” [MeSH]) OR (digital workflow) OR (digital technology) OR (computerized dentistry) OR (intraoral scan) OR (digital impression) OR (scanbody) OR (virtual design) OR (digital design) OR (cad/cam) OR (rapid prototyping) OR (monolithic) OR (full-contour)} AND
	Control {(“Dental Technology” [MeSH]) OR (conventional workflow) OR (lost-wax-technique) OR (porcelain-fused-to-metal) OR (PFM) OR (implant impression) OR (hand-layering) OR (veneering) OR (framework)} AND
	Outcome {(“Study, Feasibility” [MeSH]) OR (“Survival” [MeSH]) OR (“Success” [MeSH]) OR (“Economics” [MeSH]) OR (“Costs, Cost Analysis” [MeSH]) OR (“Esthetics, Dental” [MeSH]) OR (“Patient Satisfaction” [MeSH]) OR (feasibility) OR (efficiency) OR (esthetics) OR (patient-centered outcome)}

{((“Dental Prosthesis” [MeSH]) OR (“Crowns” [MeSH]) OR (“Dental Prosthesis, Implant-Supported” [MeSH])) OR ((crown) OR (fixed dental prosthesis) OR (fixed reconstruction) OR (dental bridge) OR (implant crown) OR (implant prosthesis) OR (implant restoration) OR (implant reconstruction))} AND {(“Computer-Aided Design” [MeSH]) OR ((digital workflow) OR (digital technology) OR (computerized dentistry) OR (intraoral scan) OR (digital impression) OR (scanbody) OR (virtual design) OR (digital design) OR (cad/cam) OR (rapid prototyping) OR (monolithic) OR (full-contour))} AND {(“Dental Technology” [MeSH) OR ((conventional workflow) OR (lost-wax-technique) OR (porcelain-fused-to-metal) OR (PFM) OR (implant impression) OR (hand-layering) OR (veneering) OR (framework))} AND {((“Study, Feasibility” [MeSH]) OR (“Survival” [MeSH]) OR (“Success” [MeSH]) OR (“Economics” [MeSH]) OR (“Costs, Cost Analysis” [MeSH]) OR (“Esthetics, Dental” [MeSH]) OR (“Patient Satisfaction” [MeSH])) OR ((feasibility) OR (efficiency) OR (patient-centered outcome))}.

Searching was also conducted as a manual search in the dental literature of the following journals until 2016–09-16: Clinical Implant Dentistry & Related Research, Clinical Oral Implants Research, European Journal of Oral Implantology, Implant Dentistry, International Journal of Oral & Maxillofacial Implants, Journal of Clinical Periodontology, Journal of Computerized Dentistry, Journal of Dental Research, Journal of Oral & Maxillofacial Surgery, Journal of Oral Implantology, Journal of Periodontal & Implant Science, Journal of Periodontology. An additional search of the bibliographies of all full-text articles, selected from the electronic search, was performed.

### Inclusion criteria

This review included randomized controlled trials (RCT) retrieved by the systematic literature search outlined above focusing on any clinical outcome with regard to complete digital workflows in fixed prosthodontics or economics as time and cost analyses or esthetics or patient-centered outcomes with or without follow-up or survival/success rate analysis as well as complication assessment of at least one year under function.

In detail, the criteria for study selection were:

- Treatment concepts with fixed prosthodontic reconstructions, tooth-borne or implant-supported for single- or multi-units;
- Processing of a complete digital workflow (without physical model situation);
- Given information on the used clinical work steps and technical production.

### Selection of studies

**Fig. 1:**
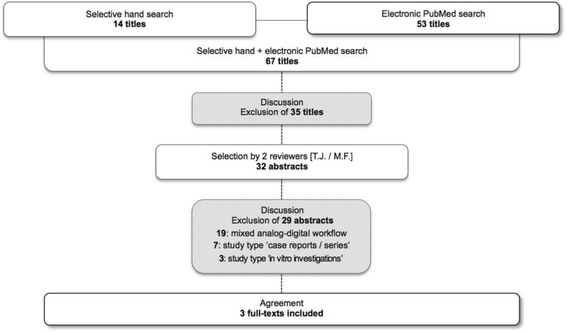
Flow-chart depicting the electronic and manual search results

Based on the defined inclusion criteria, titles and abstracts retrieved by this systematic search were independently screened by two reviewers (T.J. & M.F.). Disagreements were resolved by discussion. Following this, abstracts of all titles agreed on by both investigators were obtained and screened again for meeting the inclusion criteria. The selected articles were then obtained in full-texts. Again, disagreements were resolved by discussion [Fig. 1].

### Data extraction

The following information was obtained from the included publications: author(s), year of publication, description of the specific study design, number of patients treated and examined, type of fixed reconstruction including number of abutment teeth and/or dental implants, clinical treatment concept and methodological approach for laboratory processing, description of the material properties as well as defined primary (and secondary) outcomes. Included studies were divided into subgroups for tooth-borne reconstructions: crowns (A1) and multi-units FDPs (A2); as well as for implant-supported reconstructions: crowns (B1) and multi-unit FDPs (B2) [Table 2].

**Table 2:** General data of the three included trials: study design, type of fixed reconstruction, number of investigated subjects, and defined outcome(s)

No.	Study (year)	Study design	Type of reconstruction	Number of subjects	Outcome
1.	Batisse et al. (2014)	RCT - crossover design - double-blinded - without follow-up	A1. Tooth-borne crowns - n = 10: monolithic LS2 - *n* = 10: metal-ceramic (gold-alloy)	8 patients [10 crowns]: randomized treatment sequence	Esthetics: - patients & operators preference.
2.	Batson et al. (2014)	RCT - 3-armed design - non-blinded - without follow-up	A1. Tooth-borne crowns - n = 10: monolithic LS2 - *n* = 10: monolithic ZrO2 - n = 12: metal-ceramic (gold-alloy)	22 patients [32 crowns]: 3 randomized groups	Marginal discrepancy / precision: - micro-computed tomography; Quality of soft tissue response: - gingival crevicular fluid rates.
3.	Joda & Bragger (2016)	RCT - 2-armed design - non-blinded - without follow-up	B1. Implant-supported crowns - n = 10: monolithic LS2 - *n* = 10: ZrO2 coping veneered	20 patients [20 implant-crowns]: 2 randomized groups	Feasibility testing; Time-efficiency: - clinical & technical workflows.

*LS2* lithium disilicate, *ZrO2* zirconium dioxide

The reported results of the studies were specified according to the defined outcomes on a patient level, and if applicable, a meta-analysis was conducted. Assessment of risk of bias in individual studies was done at a ‘trial level’ including random sequence generation, allocation concealment, blinding, completeness of outcome data, selective reporting, and other bias using the Cochrane Collaboration tool (http://ohg.cochrane.org). A judgment of risk of bias was assigned if one or more key domains had a high or unclear risk of bias.

An official registration of the systematic review was not performed.

## Results

### Included studies

The systematic search was completed on 2016–09-16 and results are current as of this date. Of the 67 titles retrieved by the search, 32 abstracts were identified, and subsequently, 29 were excluded from the final analysis [Additional file 1]. The reasons for exclusion were:

- Data of a ‘mixed analog-digital workflows’ (*n* = 19);
- Data of ‘technical reports or case series’ (*n* = 7);
- Data of ‘in-vitro investigations’ (*n* = 3).

Finally, three full-texts were included for further data extraction. All studies included in this systematic review were designed as mono-centered RCTs in an institutional university setting and were published in the last 2 years; all studies were judged to be of sufficient quality [Additional file 2].

### Descriptive analysis

**Fig. 2:**
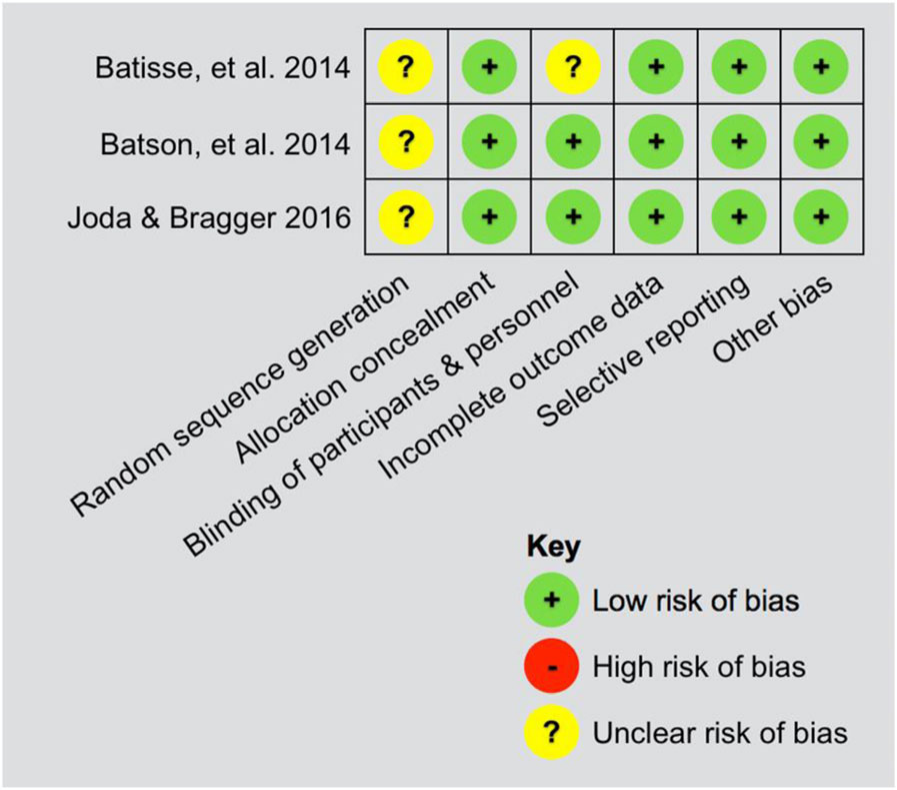
Presentation of risk of bias assessments for included studies according to the Cochrane Collaboration’s tool

Three RCTs could be selected for analysis: two studies exploring tooth-borne crowns (A1) (Batisse, et al. 2014; Batson, et al. 2014), and one study analysing implant-supported crowns (B1) (Joda & Bragger 2016) whereas no RCTs could be screened investigating multi-unit FDPs, neither tooth-borne (A2) nor implant-supported (B2). Due to the heterogeneity of the included RCTs, a direct comparison among the identified publications was not feasible, and subsequently, a meta analysis could not be performed. Therefore, the review of the full-texts followed a descriptive analysis. Detailed information of each study is shown in Tables 3 and 4. Figure 2 displays assessments of the risk of bias for the included studies. No additional analyses were performed.

**Table 3:** Detailed study information according to the type of reconstruction A1

No.	Study (year)	Number of subjects	Number of prosthetic units	Number of abutment teeth	Workflow and materials	Results
1.	Batisse et al. (2014)	*n* = 8	n = 20 [10 | 10]	*n* = 10	A1. Tooth-borne crown [maxillary incisors] Complete digital workflow (model-free) [*n* = 10] Monolithic leucite-reinforced glass ceramic (IPS Empress CAD) IOS Cerec (Sirona) + lab-software (Sirona) > Crossover study group design with randomized treatment sequence < Mixed analog-digital workflow (stone cast) [n = 10] Gold-alloy coping + hand-layered ceramic veneering Conventional impression + lost-wax-technique	Digitally produced monolithic leucite-reinforced glass ceramic crowns were esthetical favoured by the patients (8/2 crowns) and the clinicians (7/3 crowns) (*p *< 0.05). No reported follow-up time.
2.	Batson et al. (2014)	n = 20	*n* = 32 [10 + 10 + 12]	*n* = 32	A1. Tooth-borne crown [maxillary | mandible premolar + molar sites] Complete digital workflow (model-free) [*n* = 10] Monolithic LS2 (e.max CAD) IOS E4D (Planmeca E4D) + lab-software (3Shape) Mixed analog-digital workflow (CAD/CAM-model) [*n* = 10] Monolithic ZrO2 (Zenostar) IOS iTero (Aligntech) + lab-software (3Shape) Mixed analog-digital workflow (CAD/CAM-model) [*n* = 12] Gold-alloy coping + hand-layered ceramic veneering IOS iTero (Aligntech) + 1. printed coping >2. lost-wax-technique	Monolithic ZrO2 restorations (68.5 μm) showed the least amount of marginal discrepancy followed by metal-ceramic crowns (92.4 μm) and monolithic LS2 (113.8 μm) (*p* < 0.05). Average gingival crevicular fluid rates did not differ among the tested crown systems. No reported follow-up time.

*IOS* intraoral scan, *LS2* lithium disilicate, *ZrO2* zirconium dioxide

Tooth-borne crown: number of subjects, crowns and abutment teeth, prosthodontic materials including the used workflows for clinical treatment and laboratory processing as well as clinically relevant results

**Table 4:** Detailed study information according to the type of reconstruction B1

No.	Study (year)	Number of subjects	Number of prosthetic units	Number of implant abutments	Workflow and materials	Results
3.	Joda & Bragger (2016)	*n* = 20	*n* = 20 [10 | 10]	*n* = 20	B1. Implant-supported crown [maxillary | mandible premolar + molar sites] Complete digital workflow (model-free) [*n* = 10] Monolithic LS2 (e.max CAD) + Ti-base abutment (Variobase Straumann) IOS iTero (Aligntech) + lab-software (CARES Straumann) Mixed analog-digital workflow (CAD/CAM-model) [*n* = 10] ZrO2 coping + hand-layered ceramic veneering IOS iTero (Aligntech) + lab-software (CARES Straumann)	Feasibility for both workflows without need for any remakes. Total production time as the sum of laboratory plus clinical work steps was more than 2-fold faster for the complete digital workflow (75.3 min) compared to the mixed analog-digital workflow (156.6 min) (*p* < 0.05). No reported follow-up time.

*IOS* intraoral scan, *LS2* lithium disilicate, *ZrO2* zirconium dioxide

Implant-supported crown: number of subjects, reconstructions and implant abutments, prosthodontic materials including the used workflows for clinical treatment and laboratory processing as well as clinically relevant results

#### A1. Tooth-borne crown

Within the two included RCTs investigating complete digital workflows for the treatment with dental crowns, different methodological approaches, defined outcomes, and technical processing were reported (Batisse, et al. 2014; Batson, et al. 2014) [Table 3].

Batson, et al. (2014) performed a randomized 3-armed non-blinded controlled trial with 22 patients and 32 dental full crowns in posterior maxillary and mandibular sites. Group A (*n* = 10) was treated in a complete digital workflow including IOS (E4D, Planmeca, Roselle, USA) and monolithic lithium disilicate (LS2) restorations (e.max CAD, Ivoclar, Schaan. Liechtenstein), then compared to Group B (*n* = 10) and Group C (*n* = 12) with mixed analog-digital workflows based on CAD/CAM-milled model situations gathered from IOS (iTero, Cadent Aligntech, San Jose, USA) either with monolithic zirconium dioxide (ZrO2) crowns (Zenostar, Wieland, Pforzheim, Germany) or classical metal-ceramic crowns manufactured out gold-alloy-copings plus hand-layered ceramic veneering, respectively. Finally, all crowns were clinically tried-in and seated with glass ionomer cement. Six crowns were initially rejected and had to be remade (*n* = 3 metal-ceramic; *n* = 2 LS2; n = 1 ZrO2).

The participants were recalled for follow-up after 4 weeks and after 6 months. Clinical examinations included probing pocket depths, bleeding on probing, and gingival crevicular fluid rates. In addition, displacement cords were placed and conventional impressions with polyvinyl-siloxane were made of the crowns. The gathered impressions were sectioned and scanned with a micro-CT. Each crown was measured at six locations alongside the horizontal margin of the restoration.

Monolithic ZrO2 crowns showed the least amount of horizontal marginal discrepancy (68.5 μm ± 33.4) followed by metal-ceramic (92.4 μm ± 20.6), and monolithic LS2 (113.8 μm ± 43.2) (*p* < 0.05). Average gingival crevicular fluid rates did not differ among the three tested crown systems (Batson, et al. 2014).

Batisse, et al. (2014) reported on a randomized clinical crossover trial treating eight patients with ten maxillary incisor full crowns (six patients with one tooth to be rehabilitated and two patients with two teeth). Two treatment approaches were applied: a complete digital workflow with IOS (Cerec, Sirona, Bensheim, Germany) plus monolithic leucite-reinforced glass ceramic crowns (IPS Empress CAD, Ivoclar, Schaan, Lichtenstein), and a classical procedure with conventional impression-takings (polyvinyl-siloxane), stone casts, and lost-wax-technique for metal-ceramic crowns (gold-alloy coping with hand-layered veneering). All included patients received both restorations. Each crown was fixed alternatively and at random with temporary cement.

After one week, the patient and two clinicians evaluated the first crown for morphology and shape, colour, characterization, surface finish, periodontal integration, and occlusion. Subsequently, the initial crown was replaced by the second one and also left for one week with followed by esthetic and clinical evaluation as described above. Afterwards, the patients could choose which restoration they would like to keep. In general, the monolithic leucite-reinforced glass ceramic crowns were esthetical favoured, both by the patients and the clinicians (*p* < 0.05) (Batisse, et al. 2014).

#### B1. Implant-supported crown

Only one double-armed non-blinded RCT was identified investigating 20 patients each treated with one implant crown (Joda & Bragger 2016). The aim of the trial was to analyse time-efficiency by comparing a complete digital workflow processing reconstructions out of monolithic LS2 bonded to prefabricated titanium abutments without any physical models (*n* = 10) versus porcelain fused to customized ZrO2-suprastructures and hand-layered ceramic veneering in a mixed analog-digital workflow with CAD/CAM-generated models (n = 10) for the first line of therapy without follow-up. All implants (Straumann TL RN / WN, Institut Straumann AG, Basel, Switzerland) were located in premolar or molar sites with mesial and distal interproximal as well as antagonistic contacts. After capturing of the 3D–implant position with an IOS device (iTero, Cadent Aligntech, San Jose, USA), the study participants were randomly divided for treatment with the complete digital or the mixed analog-digital workflow.

All patients could be restored within two clinical appointments including IOS and seating of the implant crowns. No clinical adjustments were necessary for the digitally produced crowns, neither for interproximal nor occlusal sites. However, out of the 20 implant restorations manufactured in the mixed-analog-digital approach eight (40%) needed corrections interproximally, and six (30%) at occlusal surfaces. The mean total work time, as the sum of clinical plus laboratory work steps, was significantly different 75.3 min ± 2.1 for LS2 monolithic implant crowns and 156.6 min ± 4.6 for the porcelain fused to ZrO2-suprastructures (*p* = 0.0001) (Joda & Bragger 2016) [Table 4].

## Discussion

The trend of digitalization is an omnipresent phenomenon nowadays – in social life as well as in the dental community [3, 6]. The number of hits for the unspecific search term > *digital dentistry* < in PubMed (http://www.ncbi.nlm.nih.gov/pubmed) (2015: *n* = 621) is more than doubled compared to the results ten years ago (2005: *n* = 280).

However, the continuous progression of quantitative hits in PubMed cannot concurrently be related to an increase of significant research data. The systematic search of this review revealed that the out-most of the screened publications focused on mixed treatment concepts combining analog and digital work steps. In addition, the identified trials were classified as laboratory investigations, technical reports, and case series, respectively.

Overall, the scientific level of clinical evidence was lacking in the field of complete digital processing in fixed prosthodontics. Only three RCTs investigating single-unit restorations on teeth (Batisse, et al. 2014; Batson, et al. 2014) and implants (Joda & Bragger 2016) could be included for analysis. The study designs, follow-up periods as well as the defined outcomes were heterogeneous; and therefore, no evidence-based recommendations could be made. RCTs investigating multi-span units could not be found, neither tooth-borne nor implant-supported.

In general, RCTs do provide the best clinical evidence for generating a systematic review. Even though the number of included studies is very low, the team of authors believes that is worth to demonstrate the lack of evidence in the field of complete digital prosthodontic workflows. The industrial progress seems to be faster than the scientific evidence. This issue is an important result as well; and of high interest for the clinician who has to decide to invest and implement complete digital workflows in dental routine.

On a lower evidence level, case reports demonstrated feasibility testing of complete digital workflows for single-unit restorations [13–16] and short-span FDPs on teeth [17–19]. The number of clinical reports dealing with implant-supported reconstructions was limited to one case series on single-units [20], whereas no publication could be identified for implant FDPs.

Nevertheless, digital protocols are increasingly influencing prosthodontic treatment concepts [21]. Workflows for single-units, tooth-borne as well as implant-supported, might benefit mostly from the present digital trend. Monolithic CAD/CAM-processed restorations originated from IOS followed by a virtual design and production without the need of physical casting have to be considered in line of conventional manufacturing techniques for posterior restorations [12]. No space for storage of gypsum models is needed in this complete digital approach, and in case of a remake, a replica of the original restorations can be fast and inexpensively produced by means of rapid prototyping [4]. Therefore, the advantages of a virtual environment are obvious – even though the scientific validation is still pending.

The appropriate indication is a prerequisite and the correct application is absolutely crucial for the success of the overall therapy, and finally, for a satisfied patient. For digital processing, a teamwork approach is even more important and equally affects the clinician, the dental assistance, and the technician [22]. The complete digital workflow has the potential to become a game changer in fixed prosthodontics [7]. Major advantages might arise to reduce production costs [23], improve time-efficiency [24], and to satisfy patients’ perceptions [9] in a modernized treatment concept.

## Conclusions

Based on the results of the screened literature, it can be concluded that

- Included RCTs were heterogeneous and focused on different dental indications and outcomes comprising various study designs without follow-up for survival / success analyses.
- The overall scientific evidence in the field of complete digital workflows for the treatment with fixed prosthodontic reconstructions is extremely low: only three RCTs could be identified analysing tooth-borne crowns (*n* = 2), and implant-supported crowns (*n* = 1), respectively.
- No RCTs could be identified for multi-unit reconstructions; neither tooth-borne nor implant-supported FDPs.
- The scientific proof with high-quality trials seems to be slower than the industrial progress of available digital applications, tools, and devices.
- Further research is compellingly necessary to proof and confirm the initial results of the three included RCTs; therefore, clinical recommendations cannot be given based on these preliminary findings in the field of complete digital processing in fixed prosthodontics.
- Future trials should focus on clinical as well as economic outcomes comparing complete digital workflows to the well-investigated goldstandard with observation periods of more than one year.

## Additional files

Additional file 1: Excluded Studies [*n* = 29]. (DOCX 31 kb)

Additional file 2: Included Studies [*n* = 3]. (DOCX 28 kb)

## Abbreviations

CAD/CAM: Computer-assisted design / computer-assisted manufacturing
FDP: Fixed dental prostheses
IOS: Intraoral optical scanning
LS2: Lithium disilicate
RCT: Randomized controlled trial
ZrO2: Zirconium dioxide
