# Supplementary material for: The complete digital workflow in fixed prosthodontics: a systematic review
Source: BMC Oral Health. 2017 Sep 19;17:124. doi: 10.1186/s12903-017-0415-0 (PMC5606018; doi:10.1186/s12903-017-0415-0)
Supplement: Supplementary file 1 — Excluded Studies [n = 29]. (DOCX 31 kb) [file 12903_2017_415_MOESM1_ESM.docx]

***Additional file 1 – Excluded Studies [n = 29]***

1. Ahrberg D, Lauer HC, Ahrberg M, Weigl P. Evaluation of fit and efficiency of CAD/CAM fabricated all-ceramic restorations based on direct and indirect digitalization: a double-blinded, randomized clinical trial. Clinical Oral Investigations. 2016;20(2):291-300.
2. Bindl A, Mormann WH. Survival rate of mono-ceramic and ceramic-core CAD/CAM-generated anterior crowns over 2-5 years. Eur J Oral Sci. 2004;112(2):197-204.
3. Bindl A, Richter B, Mormann WH. Survival of ceramic computer-aided design/manufacturing crowns bonded to preparations with reduced macroretention geometry. Int J Prosthodont. 2005;18(3):219-24.
4. Boeckler AF, Lee H, Psoch A, Setz JM. Prospective observation of CAD/CAM titanium-ceramic-fixed partial dentures: 3-year follow-up. J Prosthodont. 2010;19(8):592-7.
5. Brawek PK, Wolfart S, Endres L, Kirsten A, Reich S. The clinical accuracy of single crowns exclusively fabricated by digital workflow: The comparison of two systems. Clinical Oral Investigations. 2013;17(9):2119-25.
6. Delben JA, Barao VA, Dos Santos PH, Assuncao WG. Influence of abutment type and esthetic veneering on preload maintenance of abutment screw of implant-supported crowns. J Prosthodont. 2014;23(2):134-9.
7. Derhalli M. The digitalizing of implant dentistry: a clinical evaluation of 15 patients. Compend Contin Educ Dent. 2013;34(3):192-6.
8. Gallucci GO, Grutter L, Nedir R, Bischof M, Belser UC. Esthetic outcomes with porcelain-fused-to-ceramic and all-ceramic single-implant crowns: a randomized clinical trial. Clin Oral Implants Res. 2011;22(1):62-9.
9. Giordano R 2nd. Zirconia: a proven, durable ceramic for esthetic restorations. Compend Contin Educ Dent. 2012;33(1):46-9.
10. Grohmann P, Bindl A, Hammerle C, Mehl A, Sailer I. Three-unit posterior zirconia-ceramic fixed dental prostheses (FDPs) veneered with layered and milled (CAD-on) veneering ceramics: 1-year follow-up of a randomized controlled clinical trial. Quintessence Int. 2015;46(10):871-80.
11. Joda T, Bragger U. Complete digital workflow for the production of implant-supported single-unit monolithic crowns. Clin Oral Implants Res. 2014;25(11):1304-6.
12. Joda T, Bragger U. Time-efficiency analysis comparing digital and conventional workflows for implant crowns: A prospective clinical crossover trial. Int J Oral Maxillofac Implants. 2015;30(5):1047-53.
13. Joda T, Katsoulis J, Bragger U. Clinical fitting and adjustment time for implant-supported crowns comparing digital and conventional workflows. Clin Implant Dent Relat Res. 2015, 22 Sep. doi: 10.1111/cid.12377 [Epub ahead of print].
14. Kern M, Sasse M, Wolfart S. Ten-year outcome of three-unit fixed dental prostheses made from monolithic lithium disilicate ceramic. J Am Dent Assoc. 2012;143(3):234-40.
15. Klink A, Huettig F. Complication and survival of Mark II restorations: 4-year clinical follow-up. Int J Prosthodont. 2013;26(3):272-6.
16. Kodama T. Implant-supported full-mouth reconstruction Malo Implant Bridge. J Calif Dent Assoc. 2012;40(6):497-508.
17. Lee SJ, Gallucci GO. Digital vs. conventional implant impressions: efficiency outcomes. Clin Oral Implants Res. 2013;24(1):111-5.
18. Lee SJ, Macarthur RXT, Gallucci GO. An evaluation of student and clinician perception of digital and conventional implant impressions. J Prosthet Dent. 2013;110(5):420-3.
19. Lin WS, Harris BT, Morton D. Use of implant-supported interim restorations to transfer periimplant soft tissue profiles to a milled polyurethane definitive cast. J Prosthet Dent. 2013;109(5):333-7.
20. Lin WS, Metz MJ, Pollini A, Ntounis A, Morton D. Digital data acquisition for a CAD/CAM-fabricated titanium framework and zirconium oxide restorations for an implant-supported fixed complete dental prosthesis. J Prosthet Dent. 2014;112(6):1324-9.
21. Naenni N, Bindl A, Sax C, Hammerle C, Sailer I. A randomized controlled clinical trial of 3-unit posterior zirconia-ceramic fixed dental prostheses (FDP) with layered or pressed veneering ceramics: 3-year results. J Dent. 2015;43(11):1365-70.
22. Pozzi A, Tallarico M, Mangani F, Barlattani A. Different implant impression techniques for edentulous patients treated with CAD/CAM complete-arch prostheses: a randomised controlled trial reporting data at 3 year post-loading. Eur J Oral Implantol. 2013;6(4):325-40.
23. Puri S, Parciak EC, Kattadiyil MT. Complete mouth reconstruction with implant-supported fixed partial dental prostheses fabricated with zirconia frameworks: a 4-year clinical follow-up. J Prosthet Dent. 2014;112(3):397-401.
24. Reich S, Schierz O. Chair-side generated posterior lithium disilicate crowns after 4 years. Clinical Oral Investigations. 2013;17(7):1765-72.
25. Sax C, Hammerle CH, Sailer I. 10-year clinical outcomes of fixed dental prostheses with zirconia frameworks. Int J Comput Dent. 2011;14(3):183-202.
26. Selz CF, Bogler J, Vach K, Strub JR, Guess PC. Veneered anatomically designed zirconia FDPs resulting from digital intraoral scans: Preliminary results of a prospective clinical study. J Dent. 2015;43(12):1428-35.
27. Takaba M, Tanaka S, Ishiura Y, Baba K. Implant-supported fixed dental prostheses with CAD/CAM-fabricated porcelain crown and zirconia-based framework. J Prosthodont. 2013;22(5):402-7.
28. Vanoorbeek S, Vandamme K, Lijnen I, Naert I. Computer-aided designed/computer-assisted manufactured composite resin versus ceramic single-tooth restorations: a 3-year clinical study. Int J Prosthodont. 2010;23(3):223-30.

29. Zarauz C, Valverde A, Martinez-Rus F, Hassan B, Pradies G. Clinical evaluation comparing the fit of all-ceramic crowns obtained from silicone and digital intraoral impressions. Clinical Oral Investigations. 2016;20(4):799-806.
