# Supplementary material for: The complete digital workflow in fixed prosthodontics: a systematic review
Source: BMC Oral Health. 2017 Sep 19;17:124. doi: 10.1186/s12903-017-0415-0 (PMC5606018; doi:10.1186/s12903-017-0415-0)
Supplement: Supplementary file 2 — Included Studies [n = 3]. (DOCX 28 kb) [file 12903_2017_415_MOESM2_ESM.docx]

***Additional file 2 – Included Studies [n = 3]***

1. Batisse C, Bessadet M, Decerle N, Veyrune JL, Nicolas E. Ceramo-metal crown or CAD/CAM rehabilitation: Patient and practitioner appraisal. Eur J Prosthodont Restor Dent. 2014;22(4):159-65.

2. Batson ER, Cooper LF, Duqum I, Mendonca G. Clinical outcomes of three different crown systems with CAD/CAM technology. J Prosthet Dent. 2014;112(4):770-7.

3. Joda T, Bragger U. Time-efficiency analysis of the treatment with monolithic implant crowns in a digital workflow: A randomized controlled trial. Clin Oral Implants Res. 2016, 06 Jan. doi: 10.1111/clr.12753 [Epub ahead of print].
